# MULTIMORBIDITY IN OLDER ADULTS: CAN DISEASE CLUSTER PREDICT DEPRESSION SEVERITY?

**DOI:** 10.1093/geroni/igz038.1450

**Published:** 2019-11-08

**Authors:** Jacquelyn Minahan

**Affiliations:** University of Kansas, Lawrence, Kansas, United States

## Abstract

Multimorbidity, defined as the co-occurrence of two or more chronic conditions, is positively correlated with depression severity among older adults. However, few studies have compared depression outcomes by disease cluster. To address this gap, secondary data analyses were performed using data from the National Social Life, Health, and Aging Project (NSHAP), Wave 2. For the purpose of this study, disease clusters are composed of conditions that implicate similar body systems (e.g., musculoskeletal system, cardiovascular system). Participants reported an average of 2.69 (+/- 1.97) chronic conditions. Multimorbidity and depressive symptom severity, as measured by the Center for Epidemiological Studies – Depression, Iowa Form (CES-D) were positively associated (p<0.001). Individual disease clusters, age, self-identifying as female, and lower educational attainment were predictive of depressive symptom severity (p<0.001). Findings support the necessary inclusion of social determinants (health status, gender, education, age) in the conceptualization of health and health outcomes within an aging population.

